# Effect of CELSR3 on the Cell Cycle and Apoptosis of Hepatocellular Carcinoma Cells

**DOI:** 10.7150/jca.39328

**Published:** 2020-02-21

**Authors:** Zucheng Xie, Yiwu Dang, Huayu Wu, Rongquan He, Jie Ma, Zhigang Peng, Minhua Rong, Zhekun Li, Jiapeng Yang, Yizhao Jiang, Gang Chen, Lihua Yang

**Affiliations:** 1 Department of Medical Oncology, First Affiliated Hospital of Guangxi Medical University, 6 Shuangyong Road, Nanning 530021, Guangxi Zhuang Autonomous Region, P. R. China.; 2 Department of Pathology, First Affiliated Hospital of Guangxi Medical University, 6 Shuangyong Road, Nanning 530021, Guangxi Zhuang Autonomous Region, P. R. China.; 3 Department of Cell Biology and Genetics, School of Pre-clinical Medicine, Guangxi Medical University, 22 Shuangyong Road, Nanning 530021, Guangxi Zhuang Autonomous Region, P. R. China.; 4 Research Department, Affiliated Cancer Hospital, Guangxi Medical University, 71 Hedi Road, Nanning, Guangxi Zhuang Autonomous Region 530021, P. R. China.

**Keywords:** hepatocellular carcinoma, CELSR3, cell cycle, apoptosis, prognosis

## Abstract

Cadherin EGF LAG seven-pass G-type receptor 3 (CELSR3) has been reported in cancers but its role and potential molecular mechanism in hepatocellular carcinoma (HCC) is unclear. Therefore, we aimed to investigate the clinical value and molecular mechanism of CELSR3 in HCC using an *in vitro* experiment, a meta-analysis and bioinformatics. The *in vitro* experiment determined the promoting effect of CELSR3 in the proliferation, invasion, and migration of HCC cells. CELSR3 knockout causes S-phage arrest in HCC cells. CELSR3 can also inhibit the apoptosis of HCC cells. The expression of the CELSR3 gene and protein was significantly elevated in HCC. Elevated CELSR3 was correlated to the bigger tumor size, higher pathological stage, and the worse overall survival of HCC. Methylation analysis revealed that the hypomethylation of CELSR3 regulated by DNMT1, DNMT3A, and DNMT3B may be the underlying mechanism of upregulated CELSR3. Biological enrichment analysis uncovered that the cell cycle, DNA replication, and PI3K-Akt signaling pathways were important pathways regulated by CELSR3 and its co-expressed genes in HCC. Taken together, upregulated CELSR3 is an important regulator in the progression and prognosis of HCC. The hypomethylation of CELSR3 and its regulation in the cell cycle may be the potential molecular mechanism in HCC.

## Introduction

Hepatocellular carcinoma (HCC), the most frequent type of liver tumor, is the 5^th^ leading cause of cancer-related death with an ascending incidence worldwide. There are an estimated 40,000 new cases and 30,000 estimated deaths of HCC patients according to the cancer statistics of 2019 in the United States 1. The well-known etiologies mainly include four factors: viral infection (hepatitis virus B/C), chemical toxicant (alcohol, afatoxin etc.), metabolic syndromes like nonalcoholic fatty liver disease, and hereditary disorders (hemochromatosis, Wilson disease etc.) 2-4. Clinical treatment for early HCC mainly includes surgical resection, local ablation, transarterial chemoembolization (TACE), and intra-arterial infusion chemotherapy 5-7. However, the mortality of HCC has remained very high, due primarily to the late-stage diagnosis 8-10. For advanced HCC, the molecular targeted agent sorafenib has become the first-line treatment and made the long-term survival of patients possible to some extent. Even so, with the problems of disease progression, drug-resistance, and adverse drug reaction as the therapy progresses, the urgent need for a more potent novel, targeted drug to replace sorafenib remains an increasingly prominent issue 11-15.

The underlying mechanisms of the development and progression of HCC are considered complicated and ambiguous. Over the past few years, considerable effort had been made in investigating the molecular mechanism and identifying novel biomarkers for HCC therapy. The current studies on the molecular mechanism mainly include the inflammation/ immune system, hormonal factors, oxidative stress, cancer stem cells, hypoxia, epithelial-mesenchymal transition, intracellular signaling pathways, the tumor microenvironment, and cancer metabolism 16-18. Genes have been widely reported to function in the regulation of the abovementioned underlying molecular mechanism of HCC. Therefore, it is important to investigate the role of important genes in clinical practice and their potential molecular mechanism in HCC.

Cadherin EGF LAG seven-pass G-type receptor 3 (CELSR3) belongs to the flamingo subfamily. The function of CELSR3 is mainly associated with epithelia, contact-dependent neurite growth, neuron migration, and axon guidance 19-21. CELSR3 has been found to function mainly in neurological disorders, such as Tourette disorder 22 and Hirschsprung disease 23. Regarding tumors, little is known about CELSR3. CELSR3 was previously found to correlated with small intestinal neuroendocrine tumors 24 and oral squamous cell carcinoma 25. Regarding liver cancer, scholars have reported that CELSR3 was up-regulated in tumor stellate cells 26. However, most existing studies on CELSR3 have not reported its practiced value in clinical diagnosis and treatment, remained at fundamental research. No studies have been done to date investigating the clinical value of CELSR3 and its possible molecular mechanism in HCC. Therefore, it is important to carry out this study to illuminate the relationship between CELSR3 and HCC.

In the current study, we aimed to investigate the clinical value and underlying molecular mechanism of CELSR3 in HCC using an *in vitro* experiment, a meta-analysis, and bioinformatics. We hope these works can provide novel perspectives of CELSR3 in the development and treatment of HCC.

## Materials and Methods

### Cell culture

The human hepatocyte-derived carcinoma cell line SMMC-7721 was obtained from our own laboratory. The cell line was cultured in Dulbecco's modified Eagle's medium (DMEM) media with 10% fetal bovine serum (FBS). The culture flask was placed in the environment of 37℃ and 5% carbon dioxide.

### Plasmid construction and transfection

Plasmid pSpCas9(BB)-2A-Puro (PX459) V2.0 (Plasmid #62988) was obtained from the addgene website (http://www.addgene.org). The construction was referenced from the Zhang Lab CRISPR Plasmids protocol 27. Two plasmids for CELSR3 in the SMMC-7721 cell line were constructed. The HCC cells (5×10^5^) were seeded into six‑well plates and cultured for 24 hours prior to transfection. We used Lipofectamine™ 3000 Transfection Reagent to perform the transfection in reference to the manufacturer's instructions. CELSR3 knockout efficiency was detected using quantitative reverse transcription PCR (RT-qPCR). The ACTB gene was used as the housekeeping gene for CELSR3 expression. The primer pairs applied for ACTB were as follows: 5′-CAGGCACCAGGGCGTGAT-3′ (forward) and 5′-TAGCAACGTACATGGCTGGG -3′ (reverse). The fold change of CELSR3 expression was calculated using the formula of 2^-ΔΔCt^.

### CCK8 proliferation assay

The CCK8 assay was utilized to detect the proliferation of the HCC cells. The CCK8 kit was purchased from Boster Biological Technology Co. Ltd. China. The CELSR3-transfected HCC cells were seeded into a 96-well plate in a density of 2000 cells per well. After incubating them for 24 hours, we evaluated the HCC cells' proliferation ability every six hours in a succession of five days. A time-proliferation curve was then drawn.

### Transwell assay

A transwell assay was used to estimate the migration and invasion of CELSR3-transfected HCC cells. For migration detection, 100μl of DMEM medium with 5% FBS containing 0.5×10^5^ HCC cells were added into the upper chamber of the 24-well plate, while 500μl of the same DMEM medium was supplemented in the lower chamber. Following incubation at 37℃ for 24 hours, the HCC cells were washed twice with PBS solution, fixed with methanol for 30 minutes, stained with 0.1% crystal violet solution, and then observed under a light microscope. For invasion detection, we purchased Matrigel (BD, 356234) from the Corning Incorporated, USA and diluted it with serum-free DMEM medium at a proportion of 1:8. Then, 60μl of diluted Matrigel was added into the upper chamber of the 24-well plate and incubated for an hour at 37℃. The incubated upper chamber was washed twice with DMEM and 100μl of serum-free DMEM containing 0.5×10^5^ HCC cells was added. Supplemented with 500μl DMEM with 5% FBS solution in the lower chamber, the HCC cells were incubated for 24 hours at 37℃. Then, similar to the migration detection, the HCC cells were washed with PBS solution, fixed with methanol, stained with 0.1% crystal violet, and counted under a light microscope.

### Flow cytometry assay

A flow cytometry assay was used to analyze the cell cycle and apoptosis for both the experiment and negative control groups. For the cell cycle analysis, a total of 5×10^5^ HCC cells were harvested, followed by centrifugation at a speed of 1200rpm/s for five minutes. After re-suspending and fixing the cells in 75% ethanol, we washed the cells with 1ml of PBS and stained them with PI/RNase dyestuff. Then, we incubated the cells in the dark for 15 minutes and used a flow cytometer to detect the cell cycle within thirty minutes. For the cell apoptosis analysis, 5×10^5^ HCC cells were collected, processed in centrifugation at a speed of 1200rpm/s for five minutes, and re-suspended in 50μl of 1×binding buffer. The cells were stained with 5μl of Annexin V-FITC and 10μl PI. The cells were incubated in the dark for 15 minutes at 25℃ and then added to 200μl of 1× binding buffer. The apoptosis was detected using a flow cytometer within an hour.

### Clinical HCC tissue samples

For detection of mRNA expression of CELSR3, a total of 100 patients primarily diagnosed with HCC during 2014 and 2015 were enrolled from Affiliated Cancer Hospital, Guangxi Medical University in this study. The 100 patients consist of 84 males and 16 females. The age of patients ranges from 24 to 79 years old. The fresh frozen HCC tissues and their paired adjacent normal tissues were used for RT-qPCR. For detection of protein expression of CELSR3, four tissue microarrays (LVC1504, LVC2281, LVC481, and LVC482) containing 329 HCC samples and 21 adjacent normal tissues were purchased from Guilin Fanpu Biotech of Guangxi, China. Shanghai Biochip Company, Ltd., Shanghai, China. In addition, ten formalin fixed paraffin embedded (FFPE) normal liver tissues, obtaining from the Department of Pathology, First Affiliated Hospital of the Guangxi Medical University (Nanning, Guangxi, China), were also included for detecting protein expression of CELSR3. The patients (295 males and 65 females), whose age range from 18 to 80 years old, were diagnosed during 2005 and 2009. The legitimacy of tissue resources that was used to make FFPE tissue microarray was confirmed by Pantomics, Inc. All the patients have signed the informed consent form. The research protocol was approved by the Ethics Committee of the Affiliated Cancer Hospital and First Affiliated Hospital, Guangxi Medical University (Nanning, Guangxi, China).

### RT-qPCR and tissue microarray

The gene expression of CELSR3 was tested in 100 paired HCC and para-cancerous tissues. Total RNA from HCC cells was extracted using a Takara PrimeScript RT Reagent Kit (Code: 9767) according to the manufacturer's protocols. The isolated RNA was used for complementary DNA synthesis using a First-Strand cDNA Synthesis Kit (Roche) according to the kit instructions. The primer pairs applied for CELSR3 and reference gene ACTB were as follows: CELSR3 forward, 5′- AGAGTATGCCTTGCGCATCA -3′; reverse, 5′- ACAGAAACTTGGAAGGGCGT -3′; ACTB forward, 5′- CAGGCACCAGGGCGTGAT -3′; and reverse, 5′- TAGCAACGTACATGGCTGGG -3′. The denaturation, annealing, and extension of one PCR cycle were set at 95 °C for 10 seconds, 60 °C for 20 seconds, and 72 °C for 20 seconds, respectively. The fold change of CELSR3 expression was calculated using the formula of 2^-ΔΔCt^.

The protein expression of CELSR3 was evaluated using immunochemistry in a tissue microarray. The construction of tissue microarrays and immunochemistry protocol were done as previously described 28, 29. One tissue microarray was constructed with 0.6 millimeters, single punches from formalin-fixed, paraffin-embedded core biopsies of 30 HCC patients. The CELSR3 anti-rabbit antibody (ab196625), and immunohistochemical staining reagents were purchased from Abcam Co. Ltd. Shanghai, China. The immunohistochemistry procedure followed the manufacturer's instruction. The staining intensity score was calculated as previously reported 30, 31.

### Collection of datasets and clinical information on HCC

To investigate the expression of CELSR3 in HCC samples and corresponding non-cancerous tissues, we systematically retrieved the Gene Expression Omnibus (GEO) database to collect available microarrays at a cutoff date of January 2019. The search strategies were the combination of the following key words: “hepatocellular OR liver” And “cancer OR carcinoma OR tumor” AND “mRNA OR gene.” The inclusion criteria were as follows: i) Both tumor samples and corresponding non-cancerous tissues were available in the dataset. ii) Gene expression profiles of both tumor samples and corresponding normal tissues were achieved. iii) The species is confined to homo sapiens. The mRNA counts profile and the clinical parameters of HCC were downloaded from the TCGA database. The mRNA data were normalized using log_2_ (x+1) algorithm. For the survival data, only patients with an overall survival over 90 days were included. Systematic meta-analysis was performed using the included GEO microarrays to determine the expression of CELSR3 in HCC tissues and normal tissues. The clinical significance of CESLR3 in the demographic and clinicopathological parameters was investigated using TCGA data. The prognostic value of CELSR3 was evaluated with a Kaplan-Meier curve.

### Methylation analysis

The DNA methylation profiles of CELSR3 and DNA methyltransferases (DNMT1, DNMT3A, and DNMT3B) were obtained from the TCGA database. Beta values are continuous variable ranks from zero to one, which represents the ratio of the intensity of the methylated bead type to the combined locus intensity. A higher beta value indicates a higher level of DNA methylation. The methylation of CELSR3 in HCC and adjacent normal samples was investigated, and the correlation between the DNA methylation and expression of CELSR3 was analyzed. We aimed to determine the relationship between CELSR3 and methyltransferase. Therefore, the correlation between CELSER3 and the three DNA methyltransferases was analyzed. The expression difference analysis of DNMT1, DNMT3A, and DNMT3B in high/low CELSR3 expression groups was also performed.

### Co-expressed genes and enrichment analysis

Co-expressed genes of CELSR3 might share similar biological functions in the process of HCC. As a result, we collected co-expressed genes of CELSR3 based on Spearman's correlation from the cBioPortal for Cancer Genomics portal (http://cbioportal.org), which provides a user-friendly interface for analyzing multidimensional cancer genomics data 32. CELSR3 and its most significant co-expressed genes (*p*<0.01, *q*<0.01) were used for the enrichment analysis to investigate their possible biological function. Gene ontology (GO) annotation including the biological process, cellular component, and molecular function were achieved. Moreover, the Kyoto Encyclopedia of Genes and Genomes (KEGG) database was used to acquire the crucial pathways in HCC. Then, a protein-protein interactive network was constructed for the enriched genes in the top significant KEGG pathway to identify the essential hub genes. The expression and prognostic significance of the hub genes were also investigated based on TCGA data. The correlation between CELSR3 and the hub genes was investigated using Spearman's test.

### Protein expression analysis

Protein expression of genes are the essential process for exerting biological function. Therefore, we investigated the protein expression of DNA methyltransferases and key genes in the gene network. The Human Protein Atlas (www.proteinatlas.org/pathology) is an interactive free-access database, which provides whole-genome transcriptome of the protein-coding genes of 17 major cancer types. The typical immunohistochemistry HCC images were obtained via The Pathology Atlas portal. The antibody staining was used to reflect the expression of protein.

### Statistics analysis

The statistical analysis was done using Stata version 15.0, IBM SPSS statistics version 25.0 (SPSS, Inc., Chicago, IL), R software version 3.52, and GraphPad Prism version 8 (GraphPad Software, Inc., La Jolla, CA, USA). Stata version 15.0 was used to perform the meta-analysis. A forest plot was applied to visualize the expression difference of CELSR3 in HCC and non-cancerous tissues. A *p*>0.05 or *I^2^*<50% indicates that no heterogeneity exists in the meta-analysis. Student's *t-*test was adopted if the data met Gaussian distribution, or the Mann-Whitney U test was then used. The log-rank test was used to evaluate the prognostic value of CELSR3. ClusterProfiler package in R was used to perform the enrichment analysis for the co-expressed genes. A *p*-value below 0.05 was regarded as significant.

## Results

### CELSR3 is downregulated in CELSR3 knockout cells

**Figure 1:**
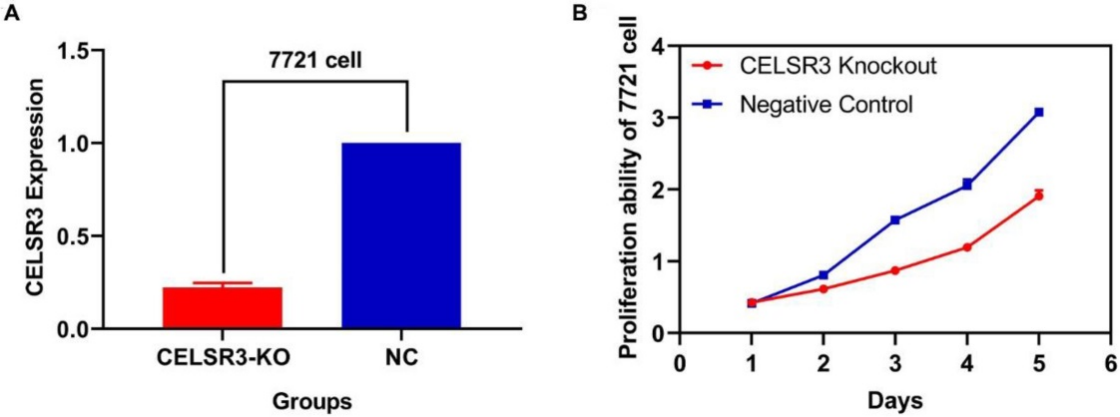
CELSR3 knockout efficacy and cell proliferation ability. A. The expression of CELSR3 in CELSR3 knockout cells was remarkably decreased. B. The proliferation ability in CELSR3 knockout cells was significantly suppressed.

Two plasmids separately lying in the upstream and downstream of the gRNA were constructed. The sequence of the two plasmids was as follows: CELSR3-E1-A (CACCGGCGTGGATCAATCTCGAAGG), CELSR3-E1-B (AAACCCTTCGAGATTGATCCACGC), among which E1 represented the first exon of gene CELSR3, while A and B represented upstream and downstream, respectively. The knockout efficacy was detected using RT-qPCR. As shown in **Figure 1A,** the expression of CELSR3 in CELSR3 knockout cells was prominently downregulated in 7721 cell lines, which confirmed the knockout efficacy.

### The correlation between CELSR3 and cells' physiological function

**Figure 2:**
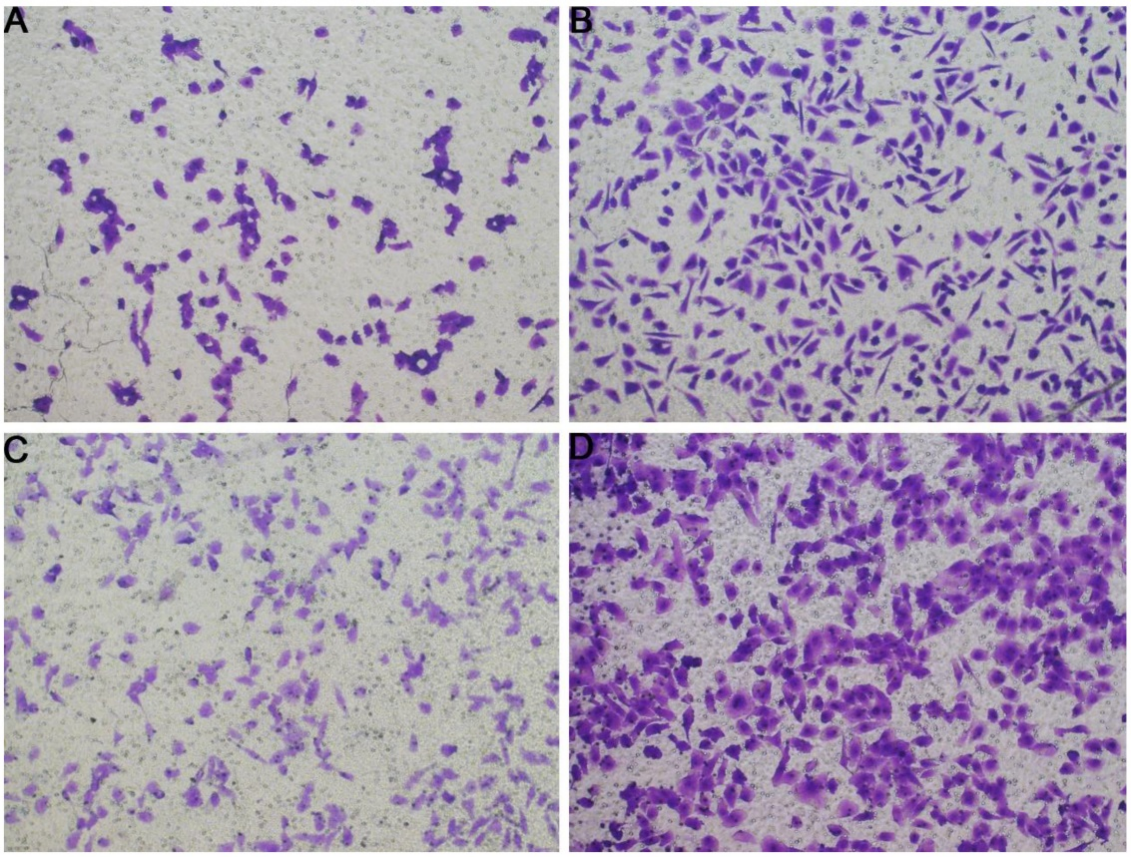
Evaluation of the migration and invasion of HCC cells. Cell migration and invasion viability were determined through the transwell experiment. A. Cell migration capacity in the CELSR3 knockout group. B. Cell migration capacity in the negative control group. C. Cell invasion capacity in the CELSR3 knockout group. D. Cell invasion capacity in the negative control group.

**Figure 3:**
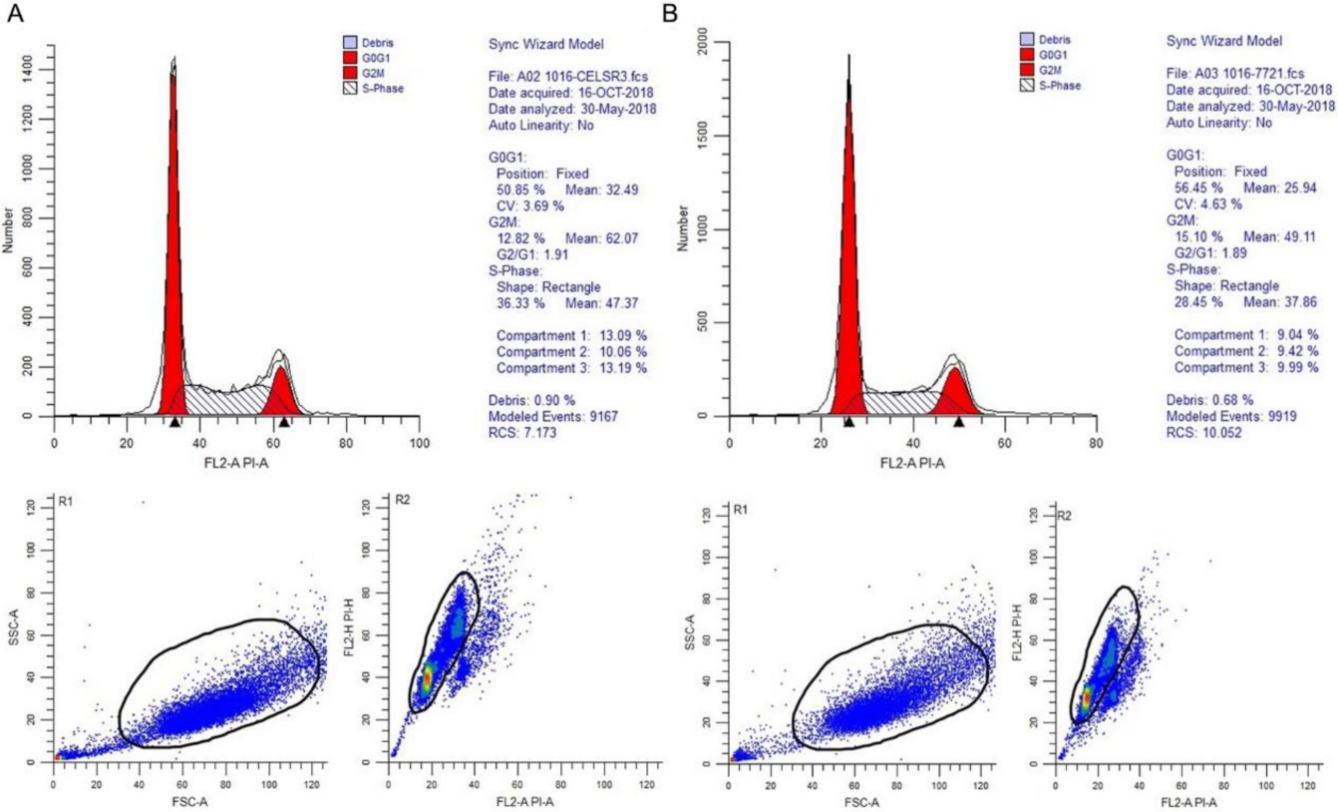
CELSR3 knockout results in S-phage arrest in HCC cells. A flow cytometry assay was utilized for the cell cycle analysis. The top part showed the DNA contents and proportion of each phage of the cell cycle. The left bottom part shows the forward and side scatter to identify the cells. The right bottom part shows the pulse area vs. height plot to identify clumps and doublets. A. CELSR3 knockout group. B. Negative control group.

**Figure 4:**
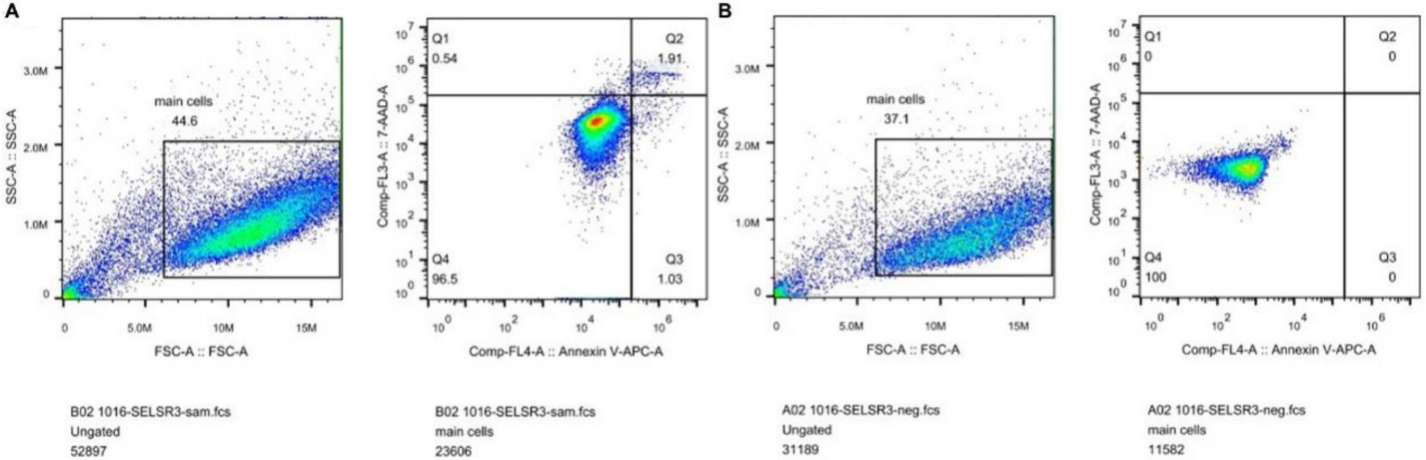
CELSR3 knockout results in the apoptosis of HCC cells. A flow cytometry assay was utilized to detect the cell apoptosis. The left part shows the forward and side scatter. The right part shows the distribution of apoptotic cells. Cells in quadrant one to four were dead, late apoptotic, early apoptotic, and live cells, respectively. A. CELSR3 knockout group. B. Negative control group.

In **Figure 1B**, the proliferation viability in the CELSR knockout group exceeded that of the negative control group, which suggested that CELSR3 was able to promote cell proliferation. The transwell assay demonstrated the migration and invasion of CELSR3 knockout cells. As shown in **Figure 2**, the migration and invasion ability were remarkably suppressed in CELSR3 knockout cells compared to the negative control cells. A flow cytometry assay was used to identify the difference in the cell cycle between CELSR knockout cells and negative control cells. The S-phage proportion in CELSR3 knockout cells was increased more than that in negative control cells (36.33% vs. 28.45%, **Figure 3**), which indicated the S-phage arrest in CELSR3 knockout cells. The apoptosis ratio in CELSR3 knockout cells was elevated compared to negative control cells (2.94% vs. 0%, **Figure 4**), suggesting the inhibition of CELSR3 in cell apoptosis.

### Expression and clinical significance of CELSR3

**Figure 5:**
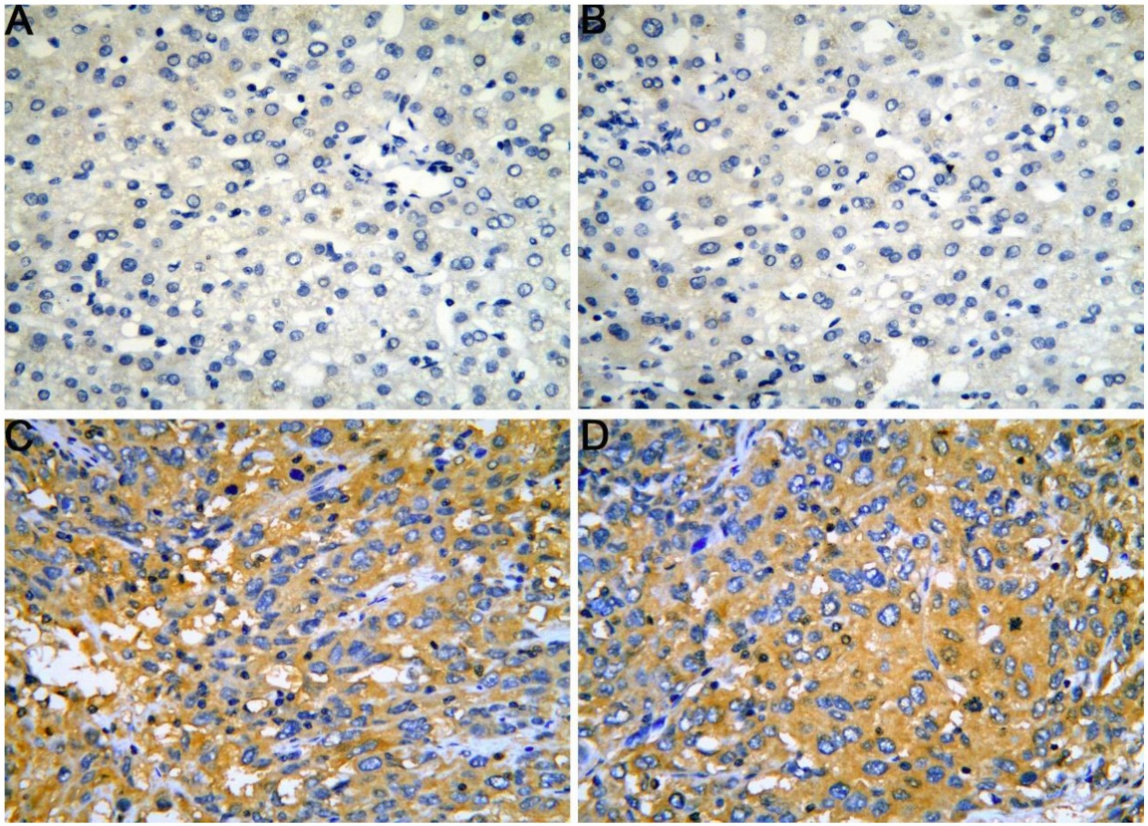
The expression of CELSR3 protein in HCC is elevated compared to adjacent normal tissues according to a tissue microarray. A-B. HCC adjacent normal tissues, magnification of 4×10. C-D. HCC tissues, magnification of 4×10.

**Figure 6:**
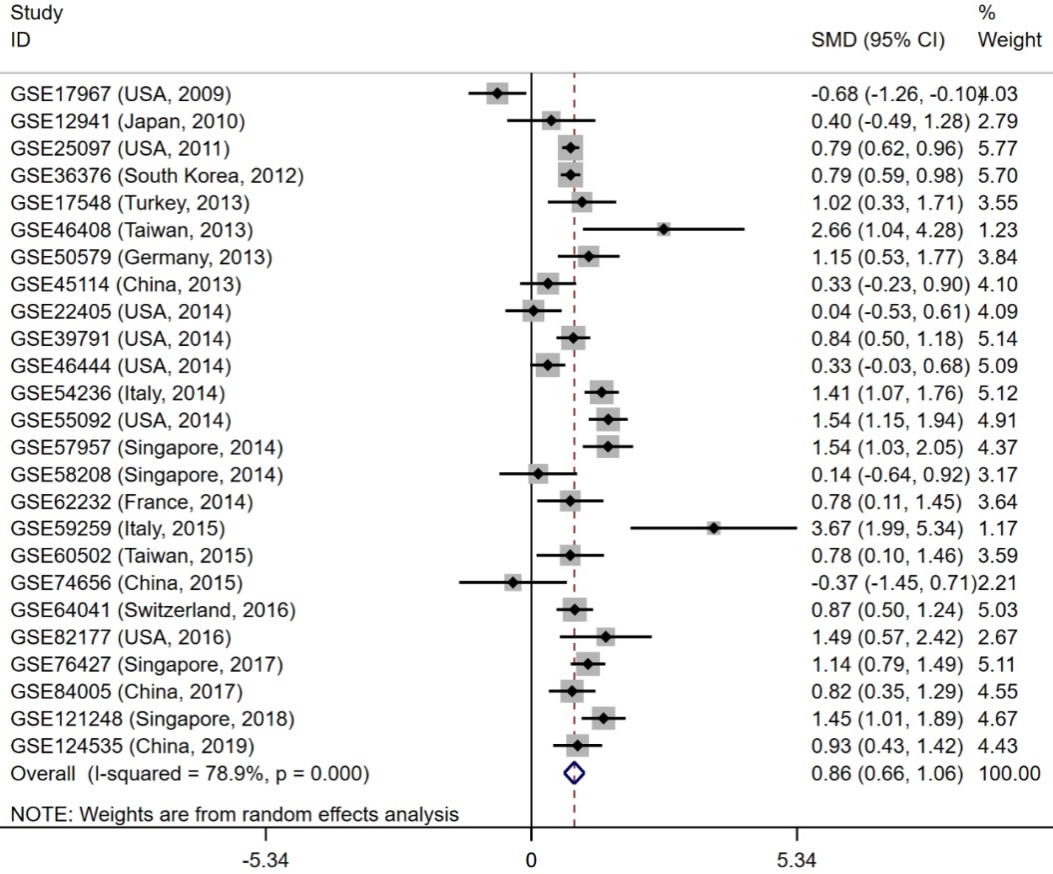
Forest plot to evaluate the expression of CELSR3 in HCC. Each dataset was displayed using a dot with the statistical weight and confidence interval. Plots at the right side of ordinate represent the high expression of CELSR3 in HCC.

**Figure 7:**
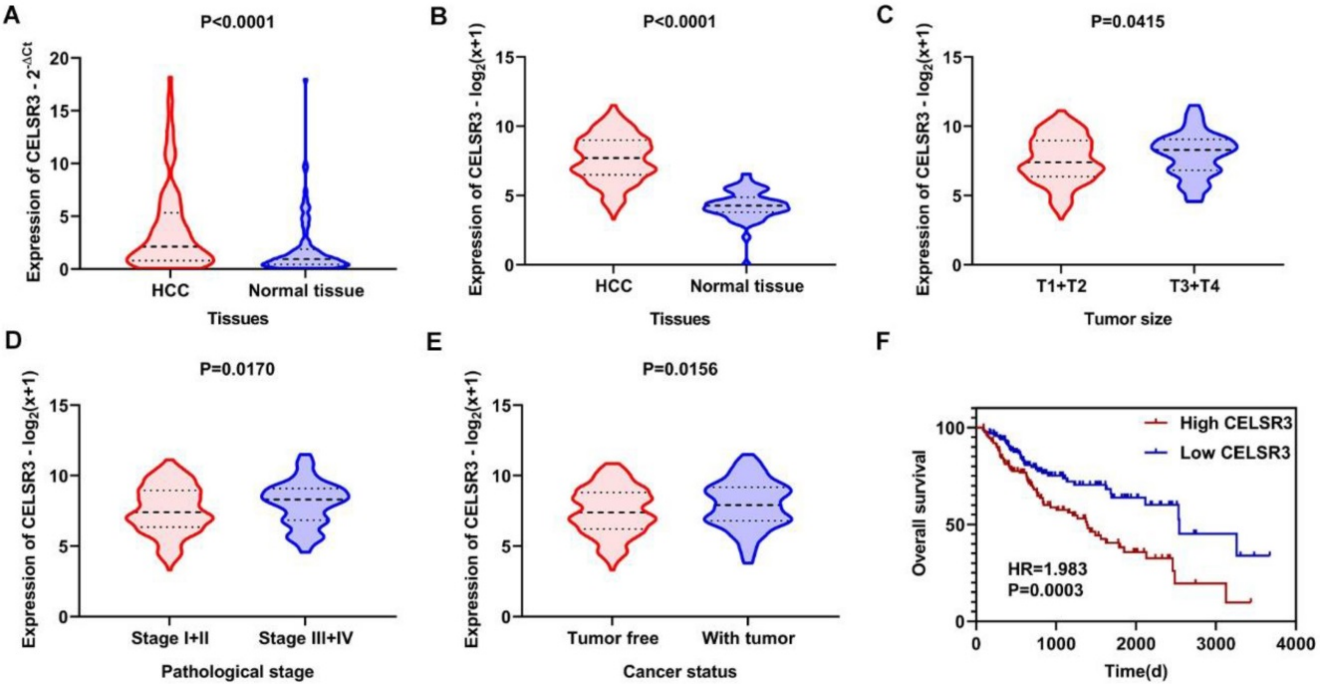
Expression and clinical significance of CELSR3 in HCC. A. The expression of CELSR3 was upregulated in HCC according to RT-qPCR. B. The expression of CELSR3 was upregulated in HCC according to TCGA data. C. The expression of CELSR3 was upregulated in larger tumor sizes. D. The expression of CELSR3 was upregulated in more advanced pathological stages. E. The expression of CELSR3 was upregulated in patients with tumors. F. High expression of CELSR3 indicated poorer survival of HCC patients.

The gene and protein expressions of CELSR3 were determined using RT-qPCR and a tissue microarray, respectively. As displayed in **Figure 7A**, the expression of CELSR3 is significantly up-regulated in HCC samples compared to adjacent normal tissues. In **Figure 5**, the immunohistochemical staining intensity in the HCC samples is much stronger than that in adjacent normal tissues, which indicated the high expression of CELSR3 protein in HCC samples. In addition, a total of 25 qualified GEO datasets containing1454 HCC samples and 1261 normal samples were included in the meta-analysis (**Table 1**). As shown in **Figure 6**, the expression of CELSR3 was upregulated in HCC compared with non-cancerous tissues. In the TCGA database, 371 HCC samples and 50 non-cancerous tissues were included. As displayed in **Table 2**, the expression of CELSR3 was validated to be upregulated in HCC samples (**Figure 7B**). Moreover, CELSR3 was found to be significantly correlated with the tumor size, pathological stage, and cancer status (**Figure 7C-7E**). A high expression of CELSR3 indicated a more advanced tumor size and pathological stage. Moreover, 330 HCC patients whose overall survival was over 90 days were included in the survival analysis. In **Figure 7F**, we can observe that a higher expression of CELSR3 indicated worse survival of HCC (HR=1.983, P=0.0003).

### Correlation of CELSR3 methylation and methyltransferases

**Figure 8:**
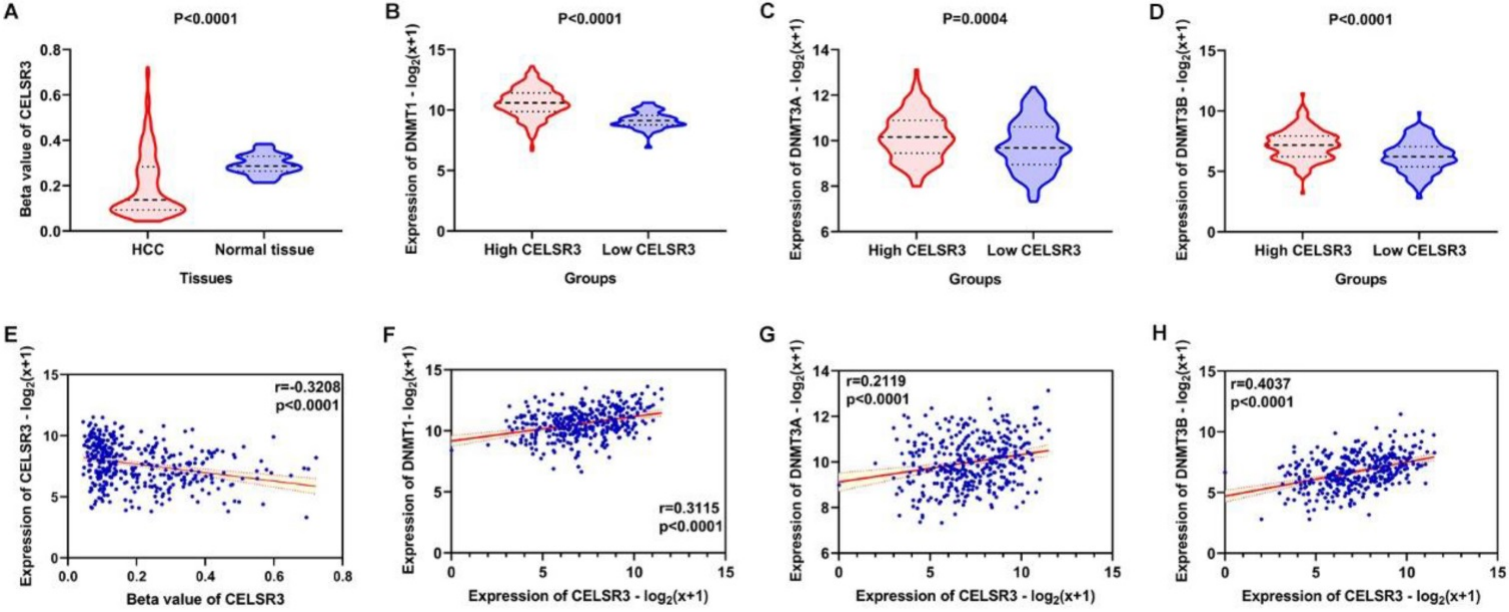
Methylation analysis of CELSR3 and methyltransferases. A. Hypomethylation of CELSR3 in HCC. B. High expression of DNMT1 in the high CELSR3 group. C. High expression of DNMT3A in the high CELSR3 group. D. High expression of DNMT3B in the high CELSR3 group. E. CELSR3 methylation was negatively correlated to CELSR3 expression. F. DNMT1 was positively correlated to CELSR3. G. DNMT3A was positively correlated to CELSR3. H. DNMT3B was positively correlated to CELSR3.

The methylation of CELSR3 in HCC samples was significantly lower than normal tissue (**Figure 8A**). CLESR3 methylation negatively correlated with the expression of CELSR3 (r=-0.3208, *p*<0.0001, **Figure 8E**). DNA methyltransferases (DNMT1, DNMT3A, and DNMT3B) are the critical enzymes catalyzing DNA methylation that exert a regulatory function in epigenetic modifications. We found that CELSR3 was positively correlated to the expression of DNMT1 (r=0.3115, *p*<0.0001), DNMT3A (r=0.2119, *p*<0.0001), and DNMT3B (r=0.4037, *p*<0.0001), respectively (**Figure 8F-8H**). We divided the HCC patients into two groups, namely high- and low-CELSR3 expression groups. We compared the expression difference of DNA methyltransferases in high/low-CELSR3 expression groups and that the expression of DNMT1, DNMT3A, and DNMT3B all significantly increased in the high-CELSR expression group (**Figure 8B-8D**).

### Enrichment analysis and protein network

**Figure 9:**
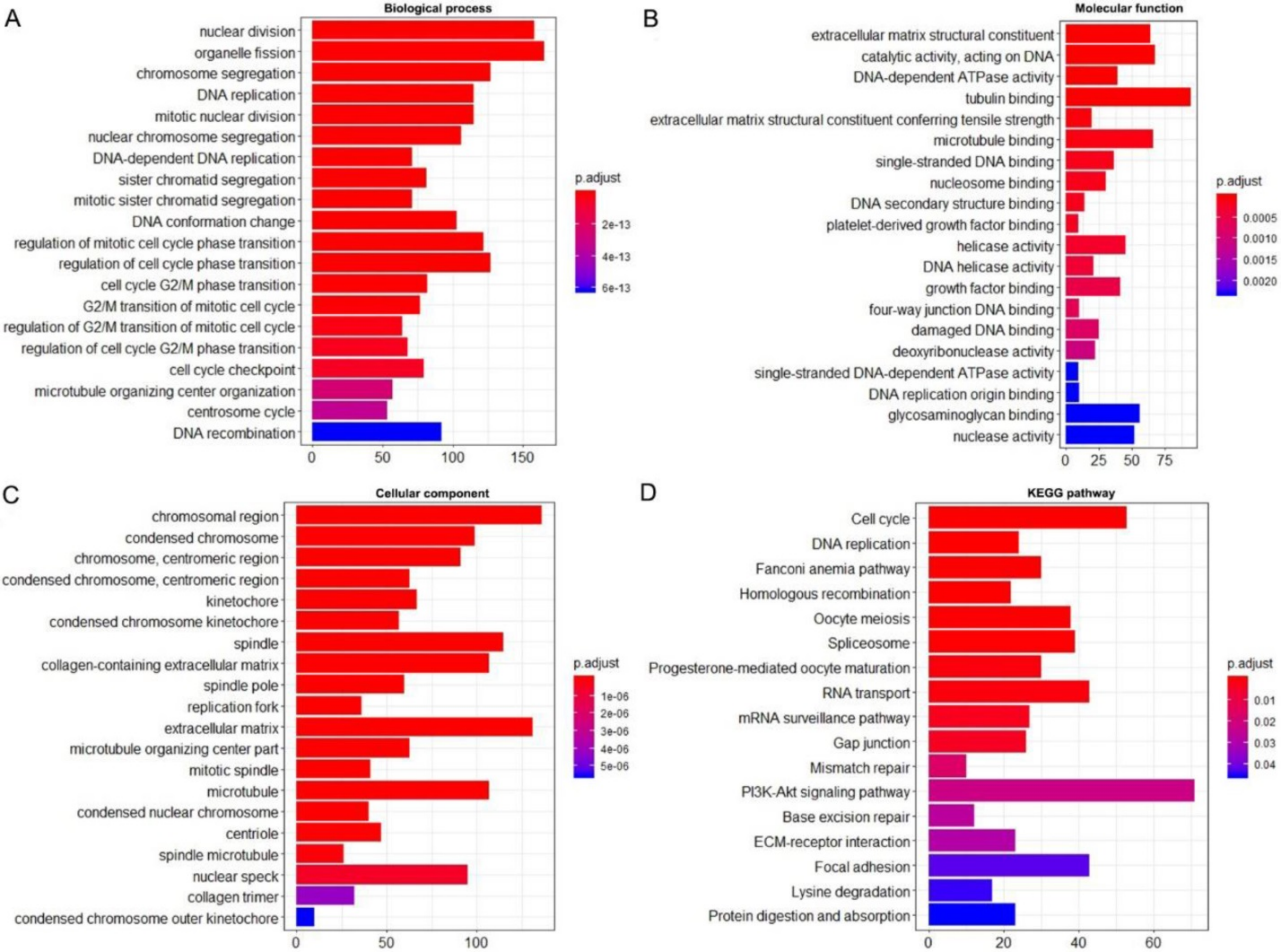
Enriched GO and KEGG terms of the co-expressed genes. A. Enriched terms for the biological process. B. Enriched terms for the molecular function. C. Enriched terms for the cellular component. D. Enriched terms for the KEGG pathway.

**Figure 10:**
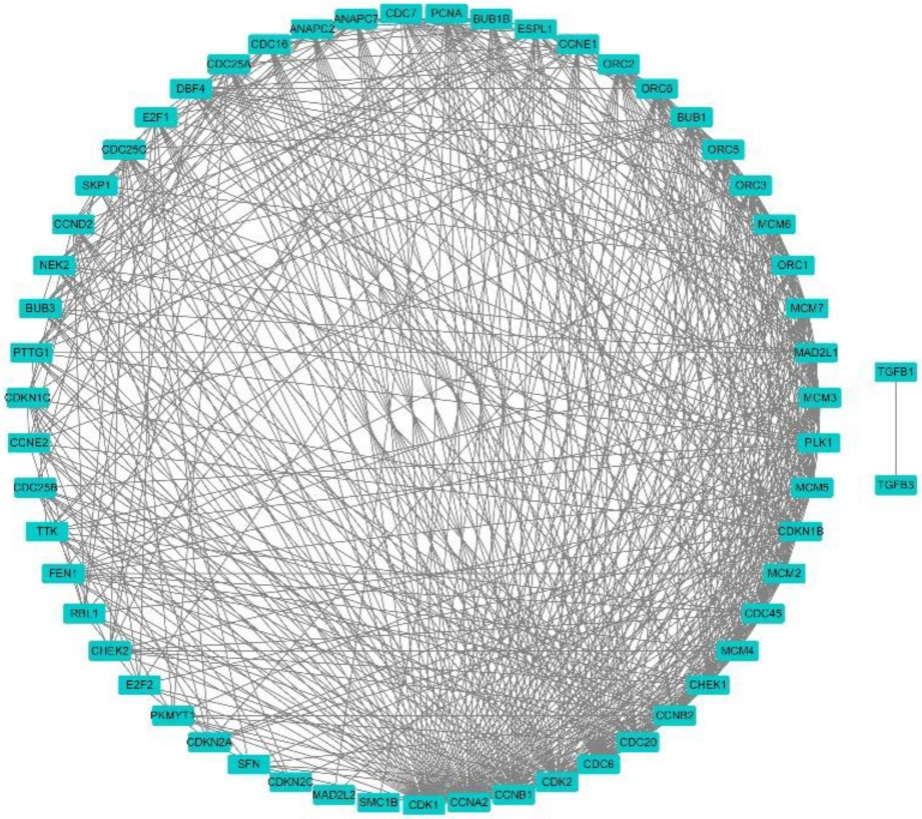
Protein-protein interactive network of co-expressed genes in the cell cycle pathway. Each node represents a gene, while the edges represent the interaction.

**Figure 11:**
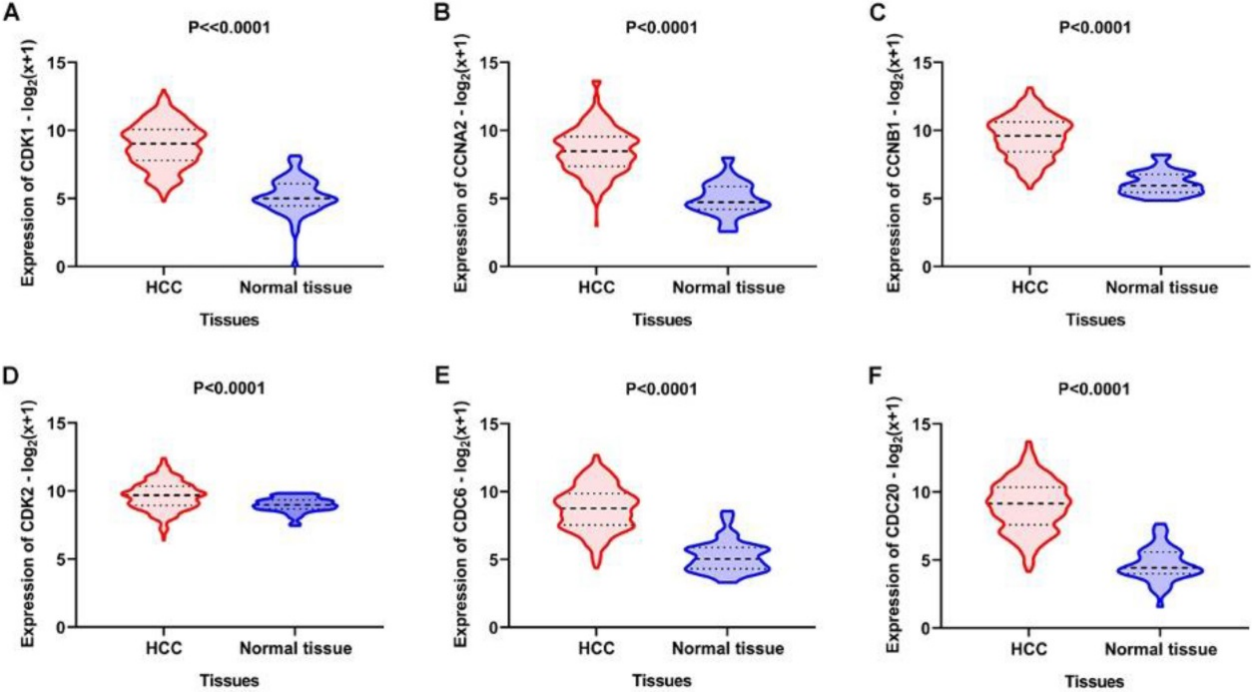
Expression of the core genes in the gene network. A-F. The expression of CDK1, CCNA2, CCNB1, CDK2, CDC6, and CDC20 was increased in HCC samples compared to normal tissues.

**Figure 12:**
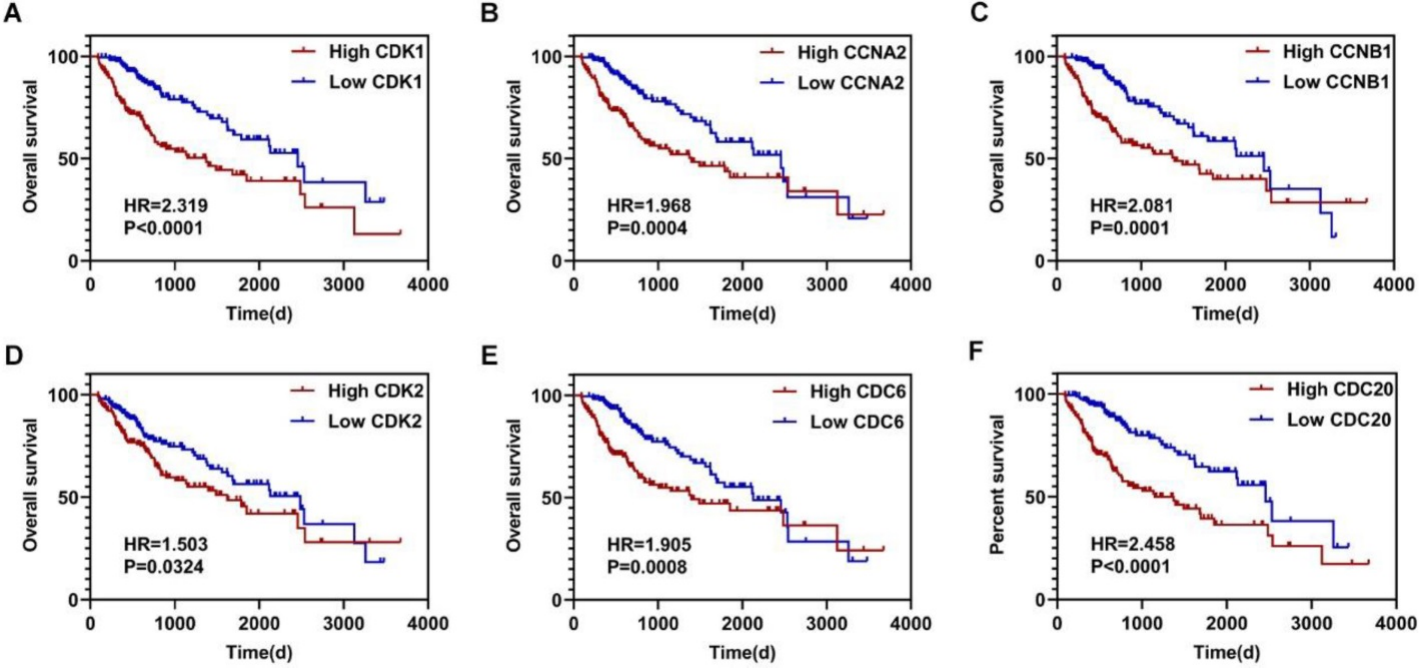
The survival curves of the hub genes in HCC. A-F. High expression of CDK1, CCNA2, CCNB1, CDK2, CDC6, and CDC20 indicate worse survival in HCC, respectively.

**Figure 13:**
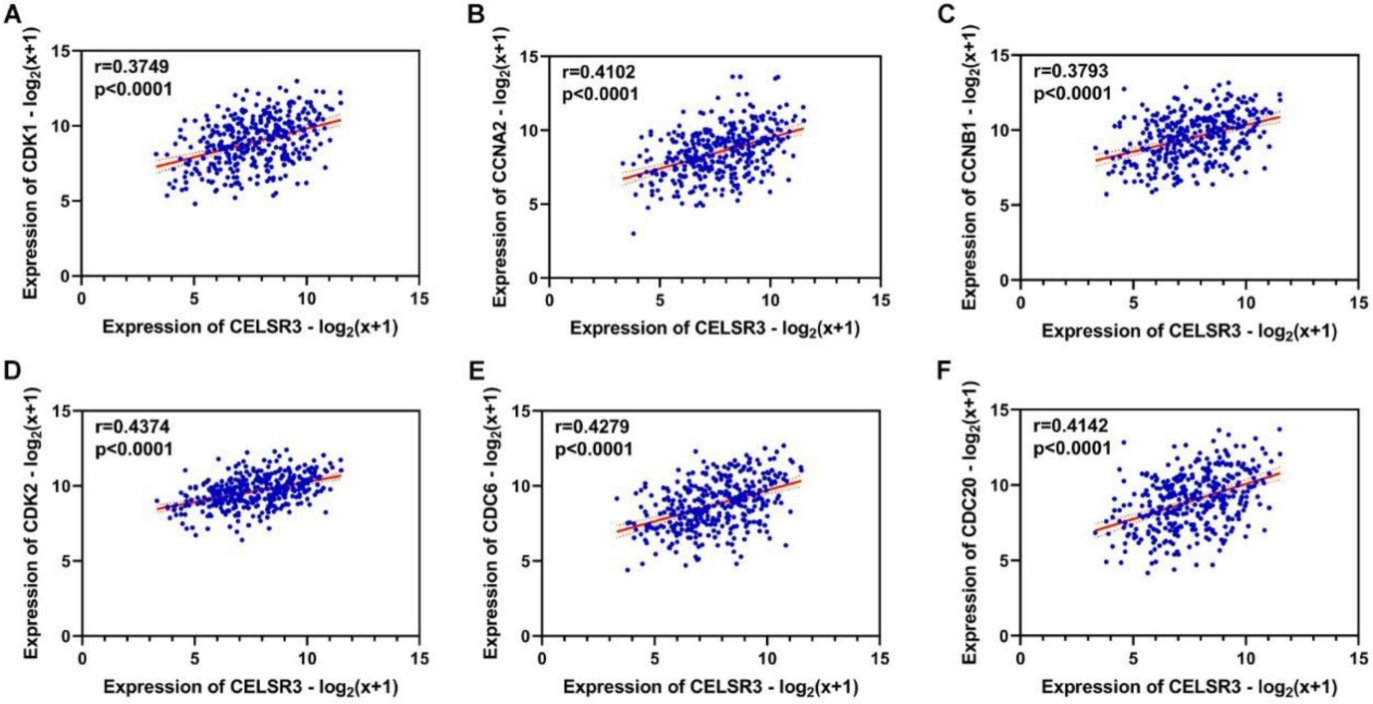
The correlation between CELSR3 and the hub genes. A-F. The expression of CDK1, CCNA2, CCNB1, CDK2, CDC6, and CDC20 was positively correlated to CELSR3, respectively.

Using the clusterProfiler package in R, the enriched terms and pathways of the co-expressed genes were identified (**Figure 9**). The cell cycle and DNA replication were the top two significantly enriched pathways. The PI3K-Akt signaling pathway was involved in the most co-expressed genes. The enriched terms in the biological process, molecular function, and cellular component were mainly related to the cell cycle and DNA replication. The enriched genes in the cell cycle pathway were visualized using a protein-protein interactive network to investigate the key hub genes. As shown in **Figure 10**, the six most connected genes with a degree over 30 (CDK1, CCNA2, CCNB1, CDK2, CDC6, and CDC20) were identified as the core hub genes in the protein-protein interactive network. The expression of the six hub genes in HCC and adjacent normal tissues were evaluated. As we can observed in **Figure 11**, the expression of CDK1, CCNA2, CCNB1, CDC6, and CDC20 was significantly upregulated in HCC samples (*p*<0.05). The prognostic value of these hub genes in HCC was further investigated. As shown in **Figure 12**, the high expression of CDK1, CCNA2, CCNB1, CDK2, CDC6, and CDC20 was associated with poor survival in HCC patients at a certain period (*p*<0.05). Moreover, the six hub genes were found to be positively correlated to CELSR3 (**Figure 13**).

### Protein expression of methyltransferases and hub genes

**Figure 14:**
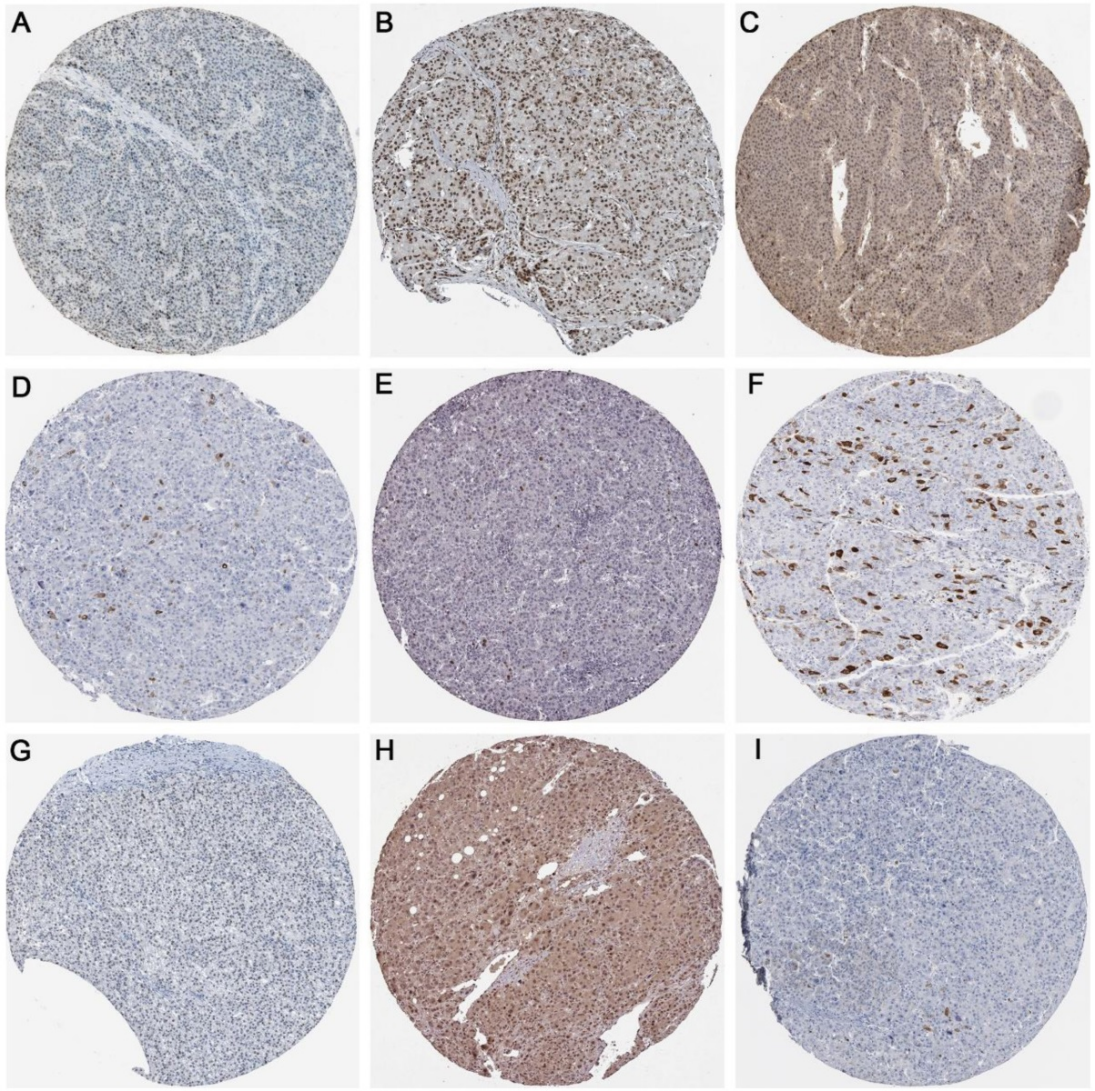
The protein expression of methyltransferases and hub genes. Immunohistochemistry staining was used to reflect the protein expression. Brown staining represents protein expression intensity. A. Moderate protein expression of DNMT1 (stained with antibody CAB005876). B. High protein expression of DNMT3A (stained with antibody HPA026588). C. Low protein expression of DNMT3B (stained with antibody HPA001595). D. Moderate protein expression of CDK1 (stained with antibody CAB003799). E. Moderate protein expression of CCNA2 (stained with antibody CAB000114). F. Moderate protein expression of CCNB1 (stained with antibody CAB003804). G. Moderate protein expression of CDK2 (stained with antibody CAB013115). H. High expression of CDC6 (stained with antibody HPA050114). I. Low expression of CDC20 (stained with antibody CAB004525).

As displayed in **Figure 14**, the protein expression of methyltransferases genes (DNMT1, DNMT3A, and DNMT3B) and hub genes (CDK1, CCNA2, CCNB1, CDK2, CDC6, and CDC20) in the protein network were investigated. The antibody staining of DNMT1, DNMT3A, and DNMT3B were medium, high and low in HCC pathological sections, respectively, which indicated moderate, high and low protein expression, separately (**Figure 14A-C**). Regarding the hub genes, the antibody staining of CDK1, CCNA2, CCNB1, and CDK2 were all moderate, suggesting moderate protein expression in HCC tissues (**Figure 14D-G**). The protein expression of CDC6 was high (**Figure 14H**), while the protein expression of CDC20 was low (**Figure 14I**).

## Discussion

HCC is a prevalent malignancy with an unfavorable outcome and ambiguous pathogenetic mechanism. Therefore, a plethora of research on the molecular mechanism and novel treatment target of HCC has recently been done. For the same reason, this study was carried out based on an *in vitro* experiment, meta-analysis, and bioinformatics analysis. We aimed to explore the clinical value and potential molecular mechanism of CELSR3 in HCC. First, we investigated the role of CELSR3 in the proliferation, invasion, migration, cell cycle, and apoptosis of HCC cells via an *in vitro* experiment. Then, we performed a meta-analysis based on the GEO datasets to determine the differential expression of CELSR3 in HCC and adjacent normal tissues. In the end, we investigated the possible molecular mechanism in HCC through bioinformatics analysis. We are the first to investigate the role of CELSR3 in HCC, hoping to provide a theoretical basis for the future therapy of HCC.

Many previous studies have unveiled important genes in HCC. For example, ZNF233 33, FOXM1 34, and CKS2 35 could promote the proliferation of HCC cells. linc-POU3F3 36, 37 RUNX2, and SLP-2 38 were found to enhance the invasion and migration of HCC cells. Regarding the prognosis- related genes of HCC, some overexpressed genes, such as SETDB1 39, SKA1^40^, and DSN1 41, were reported to be associated with poor survival in HCC. In our study, we discovered that CELSR3 could promote the cell proliferation, invasion, and migration through our *in vitro* experiment. Moreover, the apoptosis of HCC cells could be suppressed by CELSR3. We aimed to determine the expression and clinical value of CELSR3 in HCC. Hence, by virtue of the meta-analysis, we determined that the expression of CELSR3 was upregulated in HCC samples compared with adjacent normal tissues. In addition, the high expression of CELSR3 was correlated with the late T stage, advanced pathological stage, and poor survival in HCC patients. These suggested that CELSR3 was able to influence some important clinicopathological features of HCC. The results of the *in vitro* experiment might explain why CELSR3 would be associated with the T stage, pathological stage, and overall survival.

Previous studies have suggested that the methylation of genes plays an important role in hepatocarcinogenesis. For instance, GRASP overexpression controlled by methylation would significantly suppress the proliferation and invasion of HCC cells 42. Our study demonstrated that the methylation of CELSR3 was decreased in HCC and negatively correlated with the expression of CELSR3. Three types of DNA methyltransferases (DNMT1, DNMT3A, and DNMT3B) were positively correlated to the expression of CELSR3. And immunohistochemistry staining indicated the protein expression of DNMT1, DNMT3A and DNMT1 in HCC tissues, which may help validate the role of methyltransferases in HCC. The pivotal roles of DNMT1, DNMT3A, and DNMT3B in HCC have been explored by scholars. DNMT1-involved regulatory pathways, such as the DNMT1/PTEN/Akt pathways, have been found to be suppressed by miR-185, leading to the inhibition of HCC cell growth 43. The NFκB/PDL1/STAT3/ DNMT1 axis was found by Liu et al to be a new target for sorafenib-resistant HCC patients 44. Regarding DNMT3A, its ability to inhibit CpG island methylation in HCC has been reported 45. The deletion of DNMT3A could suppress HCC cell proliferation via the demethylation of PTEN promoter 46. In Wang's study, DNMT3A was a target of miR-876-5p, mediating the inhibition of HCC progression 47. DNMT3B was also a widely reported methyltransferase. For example, the DNMT3B-MEG3 axis was a regulatory target of miR-26a, which was involved in the inhibition of HCC proliferation and metastasis 48. Wu et al found that miR-29c-3p could regulate DNMT3B methylation to suppress HCC progression 49. Collectively, DNMT1, DNMT3A, and DNMT3B may be implicated in the process of CELSR3 methylation, which cause hypomethylation and high expression of CELSR3. Further experimental studies are warranted to validate the assumption.

The potential molecular mechanism of genes in the biological behavior and progress of HCC has been widely investigated, but it remains unclear. Previous studies have suggested that genes could exert a regulatory function via various signaling pathways. For example, Wang L et al discovered that CXCL17 elevates HCC cell metastasis and inhibits autophagy through the LKB1-AMPK pathway 50. In addition, Ying D et al found that MEG2 exerts suppressive functions in the growth and metastasis via inhibiting the AKT pathway 51. Our study also intended to investigate the important pathways in HCC. We discovered that the cell cycle, DNA replication, and PI3K-Akt signaling pathway were the most important pathways influenced by CELSR3 and its co-expressed genes in HCC. It is not hard to understand that the cell cycle and DNA replication pathways are pivotal mechanisms influencing cell proliferation, invasion, migration, cycle, and apoptosis. Regarding the PI3K-Akt signaling pathway, numerous studies have also reported its crucial role in the process of HCC. The PI3K-Akt signaling pathway has been widely reported to act as a key factor related to cell proliferation, invasion, metastasis, and apoptosis as well as the cell cycle 52-56. Given that, we speculated that the molecular mechanism of CELSR3 in promoting cell proliferation, invasion, and migration, as well as inhibiting apoptosis may be closely associated with the cell cycle, DNA replication, and the PI3K-Akt signaling pathway through interacting with its co-expressed genes. We further identified six important genes (CDK1, CCNA2, CCNB1, CDK2, CDC6, and CDC20) in the cell cycle pathway that may exert a regulatory function in HCC. The six hub genes are all upregulated in HCC and are associated with the prognosis of HCC patients. The expression of the six hub genes was positively correlated to CELSR3. And the majority of hub genes showed medium or high protein expression in HCC tissues. Their role in the progression of HCC has been previously reported. CDK1 has been found to be a target of other molecules, such as KPNA2, miR-378, and miR-582-5p, thus being implicated in cell proliferation and the cell cycle 57-59. Yang et al have revealed that miR-22-regulated CCNA2 repression by waltonitone was associated with the inhibition of HCC cell proliferation and tumorigenesis 60. CCNB1 has also been reported to be targeted by miR-144 to inhibit the proliferation, migration, and invasion of HCC 61. Upregulated CCNB1 was found to predict worse survival of HCC patients 62. Regarding CDK2, Lian et al found that increased CDK2 plays a synergistic role with LncRNA-MINCR in the invasion, metastasis, and poor prognosis of HCC 63. The polymorphism of CDC6 was discovered to be associated with the risk for HCC 64, 65. Increased CDC20 has also been implicated in the development, progression, and prognosis of HCC patients 62, 66. As a result, additional focus on the molecular mechanism of the six hub genes in HCC is warranted.

Several limitations needed to be addressed in the current study. First, although we provided possible mechanism of up-regulated CELSR3 and important pathways in HCC using bioinformatics data, it still lacks experimental validation. It is necessary to validate our speculations using experimental data in the future. Besides, we utilized *in vitro* experiments to validate the biological functions of CELSR3 in HCC. However, *in vivo* experiment, which has not been achieved in this study, is still a powerful method for validation of the role of CELSR3 in HCC. Research concerning CELSR3 in the future should focus more on the *in vivo* experiments.

In conclusion, we found that CELSR3 could promote HCC cell proliferation, invasion, migration, and inhibit cell apoptosis through an *in vitro* experiment. We illuminated the upregulated CELSR3 in HCC was correlated to tumor size, pathological stage, and poor survival. The hypomethylation of CELSR3 regulated by DNMT1, DNMT3A, and DNMT3B may be the cause of increased CELSR3. Bioinformatics analysis revealed that the cell cycle, DNA replication, PI3K-Akt signaling pathway, and some core genes (CDK1, CCNA2, CCNB1, CDK2, CDC6, and CDC20) may be the potential mechanism of CELSR3 in HCC. Altogether, the findings of the current study provided the clinical value and the possible underlying molecular mechanism of CELSR3 in HCC, which may be the potential therapeutic target for HCC in clinical practice.

## Figures and Tables

**Table 1:** Information on the included datasets

Dataset	Platform	Author	Year	Country	HCC samples	Normal samples
GSE17967	Affymetrix GPL571	Archer KJ et al.	2009	USA	16	47
GSE12941	Affymetrix GPL5175	Yamada T et al.	2010	Japan	10	10
GSE25097	Rosetta GPL10687	Zhang C et al.	2011	USA	268	289
GSE36376	Illumina GPL10558	Lim HY et al.	2012	South Korea	240	193
GSE17548	Affymetrix GPL570	Ozturk M et al.	2013	Turkey	17	20
GSE46408	Agilent GPL4133	Jeng Y et al.	2013	Taiwan	6	6
GSE50579	Agilent GPL14550	Geffers R et al.	2013	Germany	67	13
GSE45114	CapitalBio GPL5918	Wei L et al.	2013	China	24	25
GSE22405	Affymetrix GPL10553	Zhang HH et al.	2014	USA	24	24
GSE39791	Illumina GPL10558	Kim J et al.	2014	USA	72	72
GSE46444	Illumina GPL13369	Chen X et al.	2014	USA	88	48
GSE54236	Agilent GPL6480	Villa E et al.	2014	Italy	81	80
GSE55092	Affymetrix GPL570	Melis M et al.	2014	USA	49	91
GSE57957	Illumina GPL10558	Mah W et al.	2014	Singapore	39	39
GSE58208	Affymetrix GPL570	Hui KM et al.	2014	Singapore	10	17
GSE62232	Affymetrix GPL570	Zucman-Rossi J et al.	2014	France	81	10
GSE59259	NimbleGen GPL18451	Udali S et al.	2015	Italy	8	8
GSE60502	Affymetrix GPL96	Kao KJ et al.	2015	Taiwan	18	18
GSE74656	GeneChip GPL16043	Tao Y et al.	2015	China	10	5
GSE64041	Affymetrix GPL6244	Makowska Z et al.	2016	Switzerland	60	65
GSE82177	Illumina GPL11154	Wijetunga NA et al.	2016	USA	8	19
GSE76427	Illumina GPL10558	Grinchuk OV et al.	2017	Singapore	115	52
GSE84005	Affymetrix GPL5175	Tu X et al.	2017	China	38	38
GSE121248	Affymetrix GPL570	Wang SM et al.	2018	Singapore	70	37
GSE124535	HiSeq X Ten GPL20795	Jiang Y et al.	2019	China	35	35

**Table 2:** Correlation between CELSR3 and the clinical parameters of HCC

Variable	Groups	Number	Mean	SD	t	P value
Tissue	Tumor	371	7.680	1.680	13.901	<0.001
	Normal	50	4.280	1.096		
Gender	Male	250	7.723	1.600	0.758	0.449
	Female	121	7.582	1.837		
Age	<65	222	7.558	1.705	-1.682	0.093
	≥65	149	7.856	1.630		
T	T1+T2	275	7.573	1.694	-2.327	0.021
	T3+T4	93	8.038	1.585		
N	No	252	7.727	1.645	-0.786	0.433
	Yes	4	8.376	0.920		
M	No	266	7.688	1.610	1.583	0.115
	Yes	4	6.408	1.074		
Stage	Stage I+II	257	7.569	1.692	-2.399	0.017
	Stage III+IV	90	8.058	1.574		
Grade	Grade 1+2	232	7.593	1.655	-1.286	0.199
	Grade 3+4	134	7.827	1.721		
Residual tumor	R0	324	7.673	1.692	0.068	0.946
	R1	18	7.645	1.587		
Cancer status	Tumor free	234	7.487	1.667	-2.431	0.016
	With tumor	110	7.957	1.682		
Child grade	A	217	7.678	1.687	-0.072	0.943
	B+C	22	7.705	1.442		
AFP	Positive	114	7.396	1.667	-1.733	0.084
	Negative	149	7.754	1.648		
Primary risk factor	No	91	7.406	1.665	-1.773	0.077
	Yes	249	7.770	1.680		
