# Effect of CELSR3 on the Cell Cycle and Apoptosis of Hepatocellular Carcinoma Cells: Erratum

**DOI:** 10.7150/jca.71215

**Published:** 2022-02-17

**Authors:** Zucheng Xie, Yiwu Dang, Huayu Wu, Rongquan He, Jie Ma, Zhigang Peng, Minhua Rong, Zhekun Li, Jiapeng Yang, Yizhao Jiang, Gang Chen, Lihua Yang

**Affiliations:** 1 Department of Medical Oncology, First Affiliated Hospital of Guangxi Medical University, 6 Shuangyong Road, Nanning 530021, Guangxi Zhuang Autonomous Region, P. R. China.; 2 Department of Pathology, First Affiliated Hospital of Guangxi Medical University, 6 Shuangyong Road, Nanning 530021, Guangxi Zhuang Autonomous Region, P. R. China.; 3 Department of Cell Biology and Genetics, School of Pre-clinical Medicine, Guangxi Medical University, 22 Shuangyong Road, Nanning 530021, Guangxi Zhuang Autonomous Region, P. R. China.; 4 Research Department, Affiliated Cancer Hospital, Guangxi Medical University, 71 Hedi Road, Nanning, Guangxi Zhuang Autonomous Region 530021, P. R. China.

In our previously published paper 1, Table 1 and Figure 6 should be corrected as follows:

## Figures and Tables

**Figure 6:**
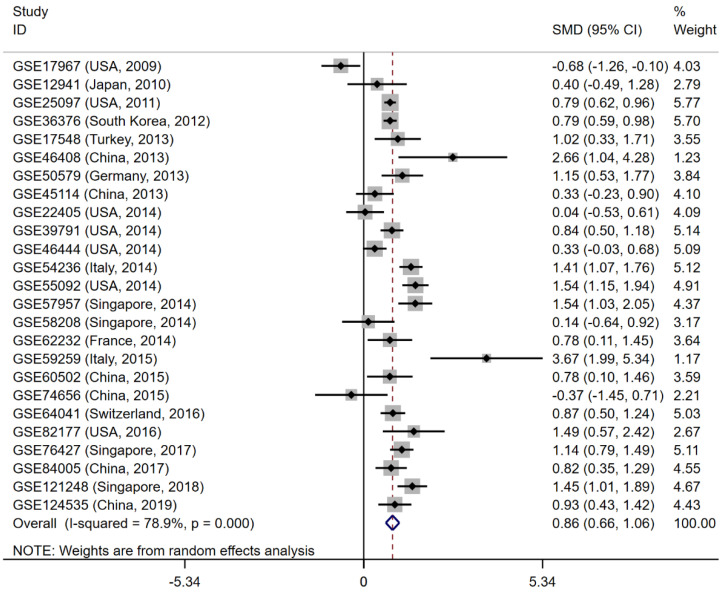
Forest plot to evaluate the expression of CELSR3 in HCC. Each dataset was displayed using a dot with the statistical weight and confidence interval. Plots at the right side of ordinate represent the high expression of CELSR3 in HCC.

**Table 1:** Information of the included datasets

Dataset	Platform	Author	Year	Country	HCC samples	Normal samples
GSE17967	Affymetrix GPL571	Archer KJ et al.	2009	USA	16	47
GSE12941	Affymetrix GPL5175	Yamada T et al.	2010	Japan	10	10
GSE25097	Rosetta GPL10687	Zhang C et al.	2011	USA	268	289
GSE36376	Illumina GPL10558	Lim HY et al.	2012	South Korea	240	193
GSE17548	Affymetrix GPL570	Ozturk M et al.	2013	Turkey	17	20
GSE46408	Agilent GPL4133	Jeng Y et al.	2013	China	6	6
GSE50579	Agilent GPL14550	Geffers R et al.	2013	Germany	67	13
GSE45114	CapitalBio GPL5918	Wei L et al.	2013	China	24	25
GSE22405	Affymetrix GPL10553	Zhang HH et al.	2014	USA	24	24
GSE39791	Illumina GPL10558	Kim J et al.	2014	USA	72	72
GSE46444	Illumina GPL13369	Chen X et al.	2014	USA	88	48
GSE54236	Agilent GPL6480	Villa E et al.	2014	Italy	81	80
GSE55092	Affymetrix GPL570	Melis M et al.	2014	USA	49	91
GSE57957	Illumina GPL10558	Mah W et al.	2014	Singapore	39	39
GSE58208	Affymetrix GPL570	Hui KM et al.	2014	Singapore	10	17
GSE62232	Affymetrix GPL570	Zucman-Rossi J et al.	2014	France	81	10
GSE59259	NimbleGen GPL18451	Udali S et al.	2015	Italy	8	8
GSE60502	Affymetrix GPL96	Kao KJ et al.	2015	China	18	18
GSE74656	GeneChip GPL16043	Tao Y et al.	2015	China	10	5
GSE64041	Affymetrix GPL6244	Makowska Z et al.	2016	Switzerland	60	65
GSE82177	Illumina GPL11154	Wijetunga NA et al.	2016	USA	8	19
GSE76427	Illumina GPL10558	Grinchuk OV et al.	2017	Singapore	115	52
GSE84005	Affymetrix GPL5175	Tu X et al.	2017	China	38	38
GSE121248	Affymetrix GPL570	Wang SM et al.	2018	Singapore	70	37
GSE124535	HiSeq X Ten GPL20795	Jiang Y et al.	2019	China	35	35
